# Epidemiology of benign essential blepharospasm: A nationwide population-based retrospective study in Taiwan

**DOI:** 10.1371/journal.pone.0209558

**Published:** 2018-12-26

**Authors:** Yng Sun, Pei-Jhen Tsai, Chin-Liang Chu, Wei-Chun Huang, Youn-Shen Bee

**Affiliations:** 1 Department of Ophthalmology, Kaohsiung Veterans General Hospital, Kaohsiung, Taiwan; 2 Kaohsiung Medical University of Hospital, Kaohsiung, Taiwan; 3 Rong Xin Mental Health Clinic, Kaohsiung, Taiwan; 4 Critical Care Center and Cardiovascular Medical Center, Kaohsiung Veterans General Hospital, Kaohsiung, Taiwan; 5 Department of Physical Therapy, Fooyin University, Kaohsiung, Taiwan; 6 School of Medicine, National Yang-Ming University, Taipei, Taiwan; 7 Yuh-Ing Junior College of Health Care and Management, Kaohsiung, Taiwan; 8 National Defense Medical Center, Taipei, Taiwan; University of Cagliari, ITALY

## Abstract

**Importance:**

This study provides a nationwide, population-based data on the incidence of benign essential blepharospasm in Asian adults.

**Background:**

To describe the incidence, patient demographics, and risk factors associated with benign essential blepharospasm.

**Design:**

Population-based retrospective study.

**Participants and samples:**

A total of 1325 patients with benign essential blepharospasm were identified.

**Methods:**

Patients with diagnosis of blepharopsasm between January 2000 and December 2013 were sampled using the Longitudinal Health Insurance Database 2000. Secondary blepharospasm that may be related to neurological, trauma, and ocular surface disease were excluded.

**Main outcome measured:**

Multivariate conditional logistic regression was used to estimate the odds ratios for potential risk factors of benign essential blepharospasm.

**Results:**

The mean annual incidence was 0.10‰ (0.07‰ for males, and 0.12‰ for females). The peak incidence was in the 50 to 59-year-old age group (0.19‰). People living in urban regions have more risk of developing blepharospasm comparing to people living in less urban regions (p <0.01). White-collar workers also have higher chance of having blepharospasm (p<0.001). Significant difference between control group and case group in hyperlipidemia (p <0.001), sleep disorders (p <0.001), mental disorders (depression, anxiety, obsessive compulsive disorder) (p <0.001), dry eye-related diseases (dry eye, Sjögren’s syndrome) (p <0.001), Parkinson’s disease (p <0.004), and rosacea (p <0.021) were also identified.

**Conclusions and relevance:**

Higher level of urbanization, white-collar work, sleep disorders, mental health diseases, dry eye-related diseases, Parkinsonism, and rosacea are possible risk factors for benign essential blepharospasm.

## Introduction

Blepharospasm is a condition characterized by excessive involuntary closure of the eyelids, generally due to spasms of the orbicularis muscles [1–4]. The most common form of blepharospasm, benign essential blepharospasm (BEB), is of unknown etiology and is considered a form of late-onset focal dystonia [4–6]. Initially, patients may experience mild and infrequent spasms. However, as symptoms progress, in 6 months to 3 years [6–8], patients may start to suffer visual disability and social withdrawal, which can significantly affect their quality of life [9].

Several factors have been associated with BEB, including female gender [10–14], onset ages between the fifth and sixth decade [10, 11], previous eye problems such as blepharitis or keratoconjunctivitis [15, 16], dry eye syndrome [16–18], and a family history of blepharospasm [15, 19, 20]. In a systematic review, Kuyper et al. investigated the relationship between dystonia (primary and other genetic forms) and non-motor symptoms of blepharospasm, including alterations in mood, cognition, sleep disturbance, autonomic function, and pain. Although the pathophysiology and neuroanatomy of these effects have not been clearly elucidated, it is thought that the underlying defects in dystonia may provide a substrate for non-motor symptoms to develop [20, 21].

There have been only a few epidemiological studies on BEB. Defazio and Livrea speculated that this may be due to the disease’s rarity and relatively low morbidity as compared to other neurological conditions [22]. Rarer yet is the investigation of BEB in Asia. Except in two studies that explored the prevalence and demographic features of BEB in select regions of Asian countries [13, 23], none has thoroughly analyzed BEB on a nationwide, populational scale. In this study, we aim to investigate patient demographics and risk factors associated with BEB in a large population of Asian descent by using data from the Taiwan National Health Insurance Research Database (NHIRD).

## Materials and methods

The National Health Insurance (NHI) program in Taiwan is a compulsory, single-payer, tax-financed healthcare system launched in 1995. It provides healthcare to 99% of the country’s 23.75 million people [24]. National Health Insurance Research Database (NHIRD) includes detailed information regarding patients’ demographic data and medical records (dates of hospital admissions and clinic visits, ICD codes, intervention procedures, prescription medications, etc.). In this study, the Longitudinal Health Insurance Database 2000 (LHID2000), a subset of NHIRD containing one million unique individuals randomly sampled between January 2000 to December 2013, was used. The Institutional Review Board of Kaohsiung Veterans General Hospital also issued a formal written waiver for patient consent specifically for this study (IRB No: VGHKS15-EM4-01).

The database was queried for blepharospasm using its ICD-9-CM (International Classification of Diseases, Ninth Revision, Clinical Modification) code, ICD-9 333.81. Incident cases were defined as persons who were diagnosed at least once with blepharospasm, both primary and secondary, at either an outpatient visit or during a hospital admission between year 2000 and 2013. Secondary blepharospasm that may be related to neurological, trauma, or ocular surface disease (one month prior to the diagnosis of BEB) were excluded via diagnostic and surgical ICD-9-CM coding. In order to involve only cases of blepharospasm that are idiopathic in origin, we set forth strict exclusion criteria to filter out possible secondary causes in our data analysis. Exclusion criteria included any known neurological deficit (brain injury, malformation, prior neurosurgery, malignancy, stroke, etc. known to alter the anatomy and physiology of the brain), head and neck trauma, and ocular surface disease. Other patient exclusion criteria included those less than 18 years of age, those who carried a diagnosis of blepharospasm prior to year 2000, and those with missing data. Cases that met the BEB definition were reviewed [1].

We analyzed this representative dataset to explore the incidence and risk factors for BEB. In addition, variables were collected based on a minimum of one outpatient visit or one hospital admission. After reviewing other epidemiologic studies on BEB and the already available data in NHIRD patient profile, we decided to included demographic profile (living location, job type), sleep disorders (sleep disturbance, cataplexy and narcolepsy and other specific disorders of sleep of non-organic origin), mental disorders (depression, anxiety, and obsessive compulsive disorder), dry eye related diseases (dry eye syndrome and Sjögren’s syndrome), Parkinsonism, and rosacea. Due to the nature of the Taiwanese national healthcare database, we could only obtain diagnostic codes specified by specialists such as neurologists for sleep disorders and ophthalmologists for dry eye related diseases. Therefore, no information on details could be derived regarding specific risk factors with regard to their diagnostic criteria. Typically, DSM V would be used by psychiatrists to assess these mental disorders, but it was impossible to ascertain in this study.

A propensity score, one-to-four matching technique, was used to match controls to the BEB group for analysis. Controls were matched on the following variables: sex, age ± 2.5 years old and outcome date ± 10 days. The pre-existing medical conditions of interest included hypertension, dyslipidemia, and diabetes mellitus documented at a minimum of two outpatient visits and one hospital admission.

## Statistical analysis

Statistical analysis was performed using SPSS software (version 18.0, SPSS Inc., Chicago, Illinois, USA). All data were expressed as mean ± standard deviation (SD) or percentage. Annual incidence and sex-age-specific mean annual incidence rates from 2000 to 2013 were calculated.

Annual incidence for blepharospasm was calculated as the number of incident cases per 1,000 person-years in a given year. For comparisons of mean annual incidences and medical histories between genders, Pearson’s x^2^ tests were applied. The independent Student’s t-tests were used to examine differences between the study group and the control group of continuous data. In terms of sociodemographic characteristics and the prevalence of medical conditions of interest, differences between the two groups were determined by Pearson’s x^2^ tests. Multivariate conditional logistic regression models (controlled for all variables listed above) were constructed by patients with blepharospasm to estimate the odds ratios (ORs) for potential risk factors of blepharospasm. To further investigate suspected risk factors and blepharospasm in association to gender, incident cases were separated into male and female groups. Statistical significance was inferred at a two-sided p value of less than 0.05.

## Results

Two thousand five hundred and thirty-nine (2539) patients with blepharospasm (ICD-9 333.81) were identified by randomized sampling over one million beneficiaries from NHIRD between 2000 and 2013. A total of 1325 out of 2539 cases were eventually categorized as BEB after excluding cases of secondary blepharospasm. Of these, 467 (35.2%) were men and 858 (64.8%) were women, with a mean age of 46.38 ± 13.18 years at the time of diagnosis.

**Fig 1:**
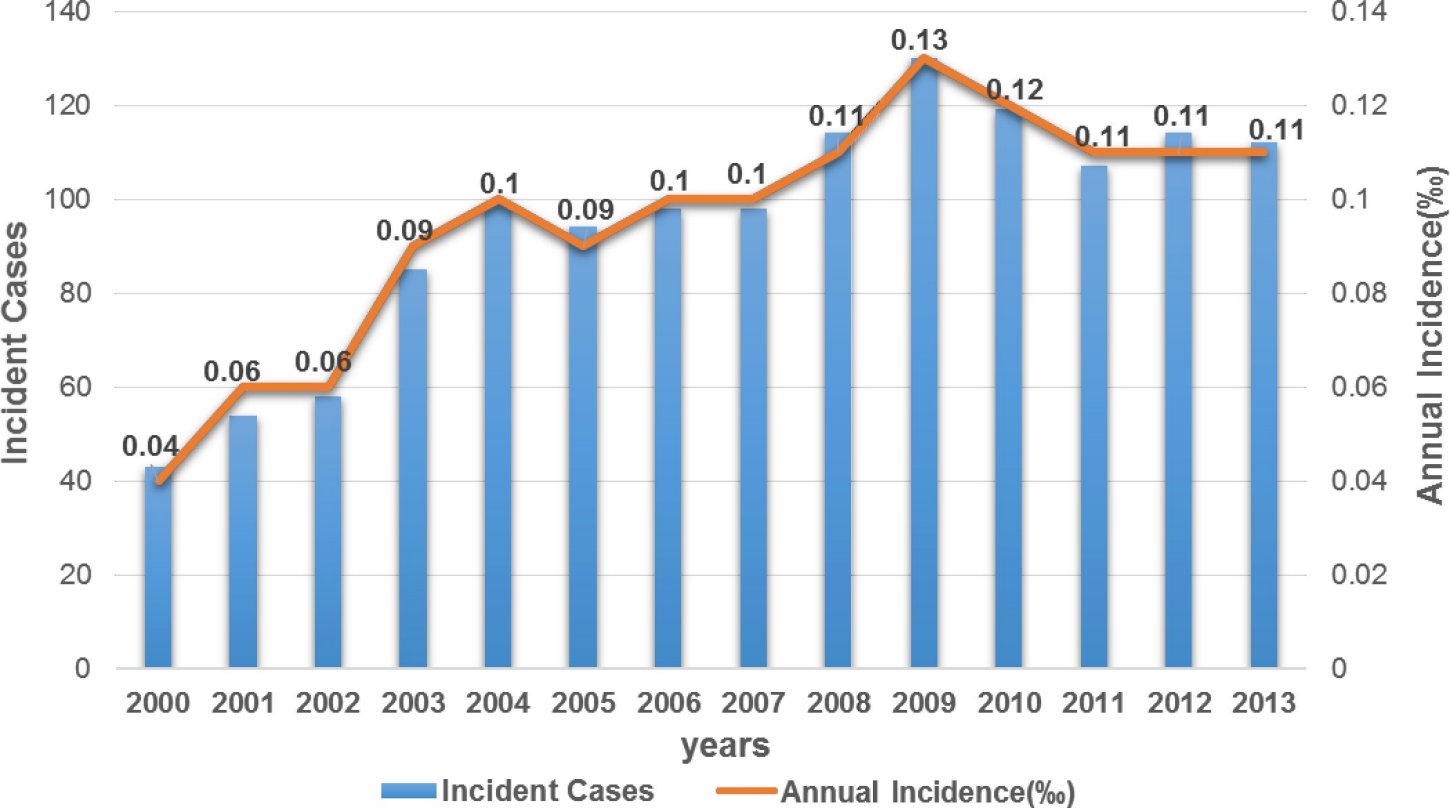
Annual incidence of benign essential blepharospasm, 2000–2013.

**Fig 2:**
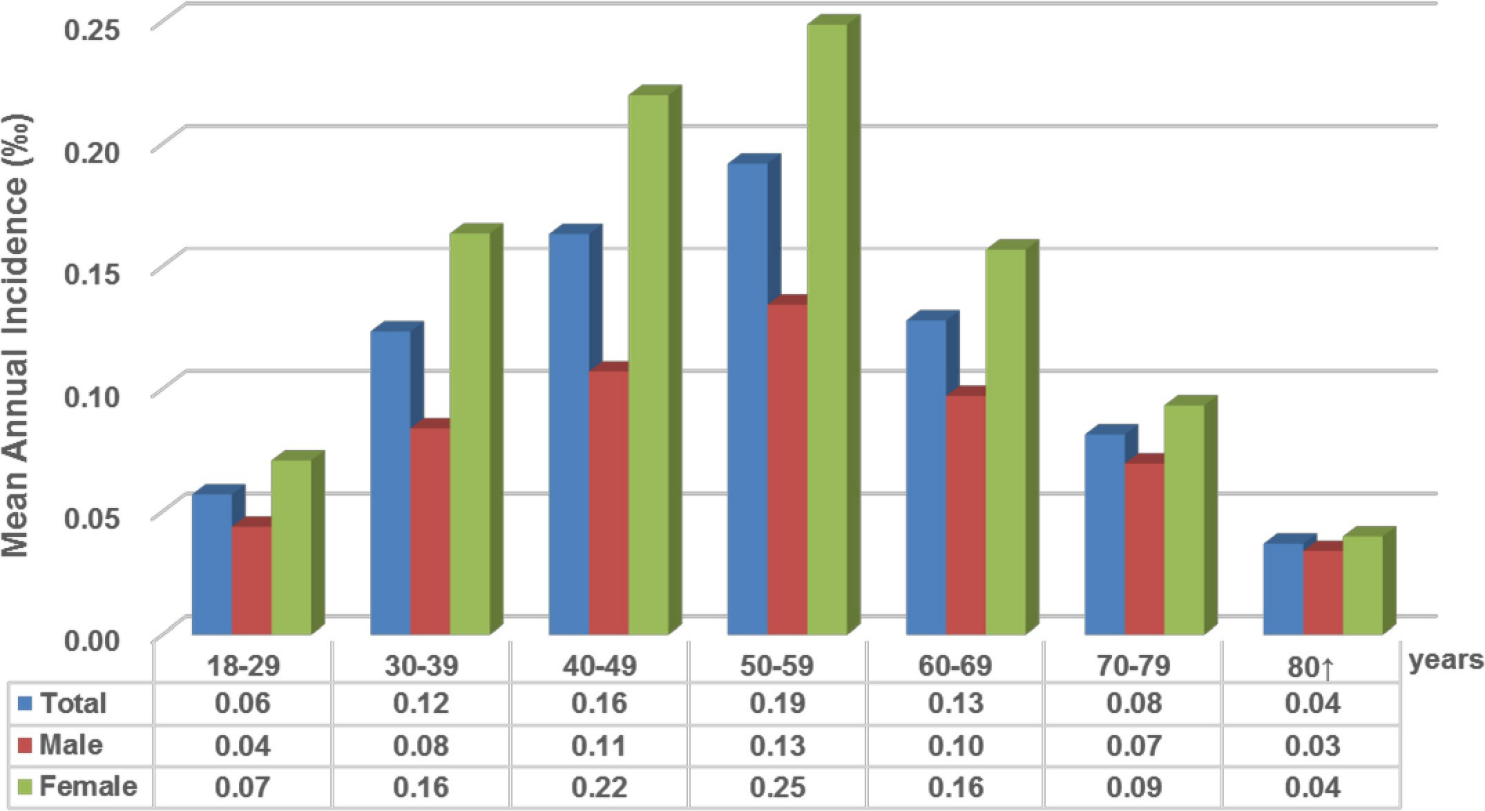
Age- and sex-specific incidence of benign essential blepharospasm, 2000–2013.

The annual incidence of BEB from 2000 to 2013 among the National Health Insurance enrollees (aged 18 and above) was listed in Fig 1. This ranged from 0.04‰ in 2000 to 0.13‰ within the next 10 years. The mean annual incidence was 0.10‰. Fig 2 lists the detailed age- and sex-specific mean annual incidence rate of the study subjects. The mean annual incidence was 0.07‰ for males and 0.12‰ for females, with a male/female ratio of 1.71. Females had a significantly higher mean annual incidence than males in all age groups. The peak mean annual incidence was in the 50–59 year age group (0.19‰), followed by the 40–49 year age group (0.13‰).

Table 1 shows the general associations between suspected risk factors and blepharospasm. Locations were divided into five different regions (north, middle, south, east and offshore islands) and had significant associations with blepharospasm (p <0.001). White-collar workers seemed to have a higher chance of developing blepharospasm (p <0.001). People with systemic diseases such as hypertension and hyperlipidemia also tended to have a higher incidence of developing blepharospasm (p <0.001). There were also significant differences between control group and case group in sleep disorders (p <0.001), mental disorders (depression, anxiety, obsessive compulsive disorder) (p <0.001), dry eye-related diseases (dry eye syndrome and Sjögren’s syndrome) (p <0.001), Parkinson’s disease (p <0.004), and rosacea (p <0.021).

**Table 1:** Demographic and characteristics of benign essential blepharospasm in comparison with control group.

	Total	Blepharospasm	Control	P value
**Factor**	**n = 6625(%)**	**n = 1325(%)**	**n = 5300(%)**	** **
**Age(mean±SD)**	**46.38±13.18**	**46.45±13.20**	**46.36±13.18**	**0.825**
**Gender (male/female)**	**2335/4290 (35.2%/64.8%)**	**467/858** **(35.2%/64.8%)**	**1868/3432** **(35.2%/64.8%)**	**1.000**
**Location**				
** Northern region**	**3480(52.5%)**	**812(61.3%)**	**2668(50.3%)**	**<0.001**
** Central region**	**1206(18.2%)**	**236(17.8%)**	**970(18.3%)**	
** Southern region**	**1686(25.4%)**	**242(18.3%)**	**1444(27.2%)**	
** Eastern region**	**139(2.1%)**	**27(2.0%)**	**112(2.1%)**	
** Offshore islands**	**114(1.7%)**	**8(0.6%)**	**106(2.0%)**	
**Job type**				
** White collar worker**	**3359(50.7%)**	**738(55.7%)**	**2621(49.5%)**	**<0.001**
** Blue collar worker**	**2098(31.7%)**	**403(30.4%)**	**1695(32.0%)**	
** Retired or no constant work**	**1168(17.6%)**	**184(13.9%)**	**984(18.6%)**	
**Hypertension**	**1198(18.1%)**	**294(22.2%)**	**904(17.1%)**	**<0.001**
**Diabetes**	**572(8.6%)**	**128(9.7%)**	**444(8.4%)**	**0.14**
**Hyperlipidemia**	**957(14.4%)**	**259(19.5%)**	**698(13.2%)**	**<0.001**
**Sleep disorders**	**1980(29.9%)**	**572(43.2%)**	**1408(26.6%)**	**<0.001**
**Psychological disorders**				
** Depression**	**359(5.4%)**	**131(9.9%)**	**228(4.3%)**	**<0.001**
** Anxiety**	**787(11.9%)**	**283(21.4%)**	**504(9.5%)**	**<0.001**
** Obsessive compulsive disorder**	**19(0.3%)**	**11(0.8%)**	**8(0.2%)**	**<0.001**
**Dry eye-related diseases**				
**Dry eye**	**265(4.0%)**	**125(9.4%)**	**140(2.6%)**	**<0.001**
**Sjogren's syndrome**	**63(1.0%)**	**25(1.9%)**	**38(0.7%)**	**<0.001**
**Parkinson's disease**	**15(0.2%)**	**8(0.6%)**	**7(0.1%)**	**0.004**
**Rosacea**	**43(0.6%)**	**15(1.1%)**	**28(0.5%)**	**0.021**

Table 2 compares the crude and adjusted odds ratios (OR) of various risk factors for patients with blepharospasm. The adjusted OR obtained with the multivariate analysis revealed that patients who live in the northern region (more urbanized region), have white-collar work, and those with hypertension, hyperlipidemia, sleep disorders, mental disorders, dry eye-related diseases, Parkinson’s disease, and rosacea may have higher odds for developing blepharospasm. To further investigate suspected risk factors and blepharospasm in association to gender, incident cases were separated into male and female groups. Tables 3, 4 and 5 demonstrate likely associations between blepharospasm and certain risk factors separated by gender (p <0.005).

**Table 2:** Crude and adjusted odds ratios for benign essential blepharospasm.

Factor	Crude OR	(95% CI)	p value	Adjusted OR	(95% CI)	p value
**Location**						
** Northern region**	**4.033**	**1.957–8.309**	**<0.001**	**4.779**	**2.264–10.088**	**<0.001**
** Central region**	**3.224**	**1.550–6.707**	**0.002**	**3.636**	**1.707–7.742**	**0.001**
** Southern region**	**2.221**	**1.609–4.614**	**0.033**	**2.721**	**1.280–5.788**	**0.009**
** Eastern region**	**3.194**	**1.389–73.43**	**0.006**	**4.019**	**1.703–9.483**	**0.001**
** Offshore islands**	**1.000**	**Reference**		**1.000**	**Reference**	
**Job type**						
** White collar worker**	**1.506**	**1.261–1.798**	**<0.001**	**1.55**	**1.288–1.865**	**<0.001**
** Blue collar worker**	**1.271**	**1.050–1.539**	**0.014**	**1.357**	**1.111–1.656**	**0.003**
** Retired or no constant work**	**1.000**	**Reference**		**1.000**	**Reference**	
**Hypertension**	**1.387**	**1.196–1.608**	**<0.001**	**1.105**	**0.930–1.314**	**0.257**
**Diabetes**	**1.17**	**0.951–1.438**	**0.137**	**0.845**	**0.666–1.071**	**0.164**
**Hyperlipidemia**	**1.602**	**1.369–1.875**	**<0.001**	**1.281**	**1.062–1.546**	**0.01**
**Sleep disorders**	**2.1**	**1.854–2.378**	**<0.001**	**1.617**	**1.407–1.857**	**<0.001**
**Psychological disorders**						
** Depression**	**2.441**	**1.951–3.053**	**<0.001**	**1.461**	**1.133–1.882**	**0.003**
** Anxiety**	**2.584**	**2.202–3.034**	**<0.001**	**1.667**	**1.385–2.007**	**<0.001**
** Obsessive compulsive disorder**	**5.538**	**2.223–13.795**	**<0.001**	**3.804**	**1.405–10.304**	**0.009**
**Dry eye-related diseases**						
** Dry eye**	**3.839**	**2.992–4.926**	**<0.001**	**3.027**	**2.324–3.944**	**<0.001**
** Sjogren's syndrome**	**2.663**	**1.602–4.427**	**<0.001**	**1.703**	**0.985–2.943**	**0.057**
**Parkinson's disease**	**4.593**	**1.663–12.689**	**0.003**	**3.317**	**1.152–9.548**	**0.026**
**Rosacea**	**2.156**	**1.148–4.048**	**0.017**	**2.064**	**1.076–3.959**	**0.029**

**Table 3:** Characteristics of male and female cases with controls.

	Male cases	Male controls		Female cases	Female controls	
	(n = 467)	(n = 1868)	p value	(n = 858)	(n = 3432)	p value
**Age(mean±SD)**						
**Location**						
** Northern region**	**271(58.0%)**	**930(49.8%)**	**0.001**	**541(63.1%)**	**1738(50.6%)**	**<0.001**
** Central region**	**86(18.4%)**	**339(18.1%)**		**150(17.5%)**	**631(18.4%)**	
** Southern region**	**100(21.4%)**	**517(27.7%)**		**142(16.6%)**	**927(27.0%)**	
** Eastern region**	**9(1.9%)**	**41(2.2%)**		**18(2.1%)**	**71(2.1%)**	
** Offshore islands**	**1(0.2%)**	**41(2.2%)**		**7(0.8%)**	**65(1.9%)**	
**Job type**						
** White collar worker**	**272(58.2%)**	**976(52.2%)**	**0.068**	**466(54.3%)**	**1645(47.9%)**	**<0.001**
** Blue collar worker**	**117(25.1%)**	**533(28.5%)**		**286(33.3%)**	**1162(33.9%)**	
** Retired or no constant work**	**78(16.7%)**	**359(19.2%)**		**106(12.4%)**	**625(18.2%)**	
**Hypertension**	**112(24.0%)**	**350(18.7%)**	**0.011**	**182(21.2%)**	**554(16.1%)**	**<0.001**
**Diabetes**	**49(10.5%)**	**156(8.4%)**	**0.17**	**79(9.2%)**	**288(8.4%)**	**0.453**
**Hyperlipidemia**	**109(23.3%)**	**246(13.2%)**	**<0.001**	**150(17.5%)**	**452(13.2%)**	**0.001**
**Sleep disorders**	**169(36.2%)**	**339(18.1%)**	**<0.001**	**403(47.0%)**	**1069(31.1%)**	**<0.001**
**Psychological disorders**						
** Depression**	**33(7.1%)**	**53(2.8%)**	**<0.001**	**98(11.4%)**	**175(5.1%)**	**<0.001**
** Anxiety**	**83(17.8%)**	**126(6.7%)**	**<0.001**	**200(23.3%)**	**378(11.0%)**	**<0.001**
** Obsessive compulsive disorder**	**4(0.9%)**	**4(0.2%)**	**0.056**	**7(0.8%)**	**4(0.1%)**	**0.002**
**Dry eye-related diseases**						
** Dry eye**	**30(6.4%)**	**27(1.4%)**	**<0.001**	**95(11.1%)**	**113(3.3%)**	**<0.001**
** Sjogren's syndrome**	**2(0.4%)**	**8(0.4%)**	**1.000**	**23(2.7%)**	**30(0.9%)**	**<0.001**
**Parkinson's disease**	**1(0.2%)**	**2(0.1%)**	**1.000**	**7(0.8%)**	**5(0.1%)**	**0.004**
**Rosacea**	**3(0.6%)**	**6(0.3%)**	**0.395**	**12(1.4%)**	**22(0.6%)**	**0.032**

**Table 4:** Crude and adjusted odds ratios for benign essential blepharospasm among male cases and controls.

Factor	Crude OR	(95% CI)	p value	Adjusted OR	(95% CI)	p value
**Location**						
** Northern region**	**11.947**	**1.636–87.255**	**0.014**	**13.428**	**1.745–103.354**	**0.013**
** Central region**	**10.401**	**1.411–76.685**	**0.022**	**11.702**	**1.509–90.738**	**0.019**
** Southern region**	**7.930**	**1.078–58.320**	**0.042**	**9.300**	**1.201–72.012**	**0.033**
** Eastern region**	**9.000**	**1.090–74.297**	**0.041**	**10.925**	**1.254–95.147**	**0.030**
** Offshore islands**	**1.000**	**Reference**		**1.000**	**Reference**	
**Job type**						
** White collar worker**	**1.283**	**0.970–1.696**	**0.081**	**1.235**	**0.921–1.656**	**0.159**
** Blue collar worker**	**1.010**	**0.736–1.386**	**0.949**	**0.962**	**0.689–1.343**	**0.820**
** Retired or no constant work**	**1.000**	**Reference**		**1.000**	**Reference**	
**Hypertension**	**1.368**	**1.074–1.743**	**0.011**	**0.895**	**0.673–1.191**	**0.447**
**Diabetes**	**1.286**	**0.917–1.804**	**0.144**	**0.885**	**0.600–1.304**	**0.536**
**Hyperlipidemia**	**2.008**	**1.559–2.585**	**<0.001**	**1.749**	**1.295–2.361**	**<0.001**
**Sleep disorders**	**2.558**	**2.048–3.195**	**<0.001**	**2.040**	**1.598–2.605**	**<0.001**
**Psychological disorders**						
** Depression**	**2.604**	**1.665–4.072**	**<0.001**	**1.376**	**0.823–2.300**	**0.223**
** Anxiety**	**2.988**	**2.218–4.027**	**<0.001**	**1.970**	**1.399–2.774**	**<0.001**
** Obsessive compulsive disorder**	**4.026**	**1.003–16.158**	**0.049**	**2.274**	**0.496–10.424**	**0.290**
**Dry eye-related diseases**						
** Dry eye**	**4.681**	**2.754–7.955**	**<0.001**	**3.611**	**2.026–6.435**	**<0.001**
** Sjogren's syndrome**	**1.000**	**0.212–4.725**	**1.000**	**0.642**	**0.130–3.179**	**0.587**
**Parkinson's disease**	**2.002**	**0.181–22.127**	**0.571**	**1.873**	**0.155–22.702**	**0.622**
**Rosacea**	**2.006**	**0.500–8.053**	**0.326**	**1.713**	**0.418–7.020**	**0.454**

**Table 5:** Crude and adjusted odds ratios for benign essential blepharospasm among female cases and controls.

Factor	Crude OR	(95% CI)	p value	Adjusted OR	(95% CI)	p value
**Location**						
** Northern region**	**2.89**	**1.318–6.341**	**0.008**	**3.618**	**1.598–8.191**	**0.002**
** Central region**	**2.207**	**0.992–4.911**	**0.052**	**2.578**	**1.124–5.913**	**0.025**
** Southern region**	**1.422**	**0.639–3.164**	**0.388**	**1.793**	**0.782–4.108**	**0.168**
** Eastern region**	**2.354**	**0.924–6.000**	**0.073**	**3.081**	**1.170–8.111**	**0.023**
** Offshore islands**	**1.000**	**Reference**		**1.000**	**Reference**	
**Job type**						
** White collar worker**	**1.670**	**1.327–2.103**	**<0.001**	**1.769**	**1.391–2.250**	**<0.001**
** Blue collar worker**	**1.451**	**1.138–1.851**	**0.003**	**1.661**	**1.287–2.142**	**<0.001**
** Retired or no constant work**	**1.000**	**Reference**		**1.000**	**Reference**	
**Hypertension**	**1.399**	**1.160–1.687**	**<0.001**	**1.210**	**0.970–1.509**	**0.091**
**Diabetes**	**1.107**	**0.853–1.437**	**0.445**	**0.836**	**0.619–1.130**	**0.244**
**Hyperlipidemia**	**1.397**	**1.141–1.710**	**0.001**	**1.052**	**0.825–1.342**	**0.682**
**Total sleep disorders**	**1.958**	**1.681–2.280**	**<0.001**	**1.504**	**1.268–1.784**	**<0.001**
**Psychological disorders**						
** Depression**	**2.400**	**1.851–3.111**	**<0.001**	**1.538**	**1.145–2.065**	**0.004**
** Anxiety**	**2.456**	**2.029–2.972**	**<0.001**	**1.614**	**1.291–2.017**	**<0.001**
** Obsessive compulsive disorder**	**7.049**	**2.059–24.136**	**0.002**	**5.856**	**1.450–23.648**	**0.013**
**Dry eye-related diseases**						
** Dry eye**	**3.657**	**2.753–4.858**	**<0.001**	**3.004**	**2.224–4.057**	**<0.001**
** Sjogren's syndrome**	**3.124**	**1.805–5.406**	**<0.001**	**2.058**	**1.137–3.723**	**0.017**
**Parkinson's disease**	**5.638**	**1.785–17.807**	**0.003**	**4.214**	**1.259–14.107**	**0.020**
**Rosacea**	**2.199**	**1.084–4.460**	**0.029**	**2.174**	**1.040–4.547**	**0.039**

## Discussion

This population-based study estimates that the annual incidence of BEB is 10 cases per 100,000 per year in Taiwan, similar to the 16–133 cases per million reported worldwide [25]. To our knowledge, this is the first study to fully investigate the patient demographics and risk factors for BEB in a large population of Asian descent. Age distribution, female predominance, and exacerbating factors were similar to those reported in the literature [21, 23, 26, 27], with additional risk factors associated with BEB found in levels of urbanization, types of jobs, hypertension, and hyperlipidemia which were not previously reported.

The annual incidence of BEB ranged from 0.04 ‰ in 2010 then increased to 0.13 ‰ within the next 10 years. This finding is similar to those reported in other countries by Macerollo et al. [28], which demonstrates improved education of both patients and doctors over time in regards to BEB. Due to the rare nature of blepharospasm, diagnosis of the disease is often delayed for years [28]. However, Marcerollo et al. also noticed that, over time, knowledge improvement and education can lead to a shorter delay in, and increased number of, the correct diagnosis [28]. Clinically, a similar trend was noted by us with shorter delays in the diagnosis of BEB and an increased BEB diagnosis over time–we hope that with improved knowledge and awareness of the general public and medical practitioners on BEB, more people with this disease will be treated properly and promptly.

As underlined by previous prevalence studies, blepharospasm occurs mainly during the fifth to sixth decade with a female predominance [8]. The same was observed in this study, with a peak age of diagnosis at about 40 to 60 years old in both sexes (female greater than male).

Several other factors have been hypothesized to play a role in blepharospasm, but only a few have been assessed using epidemiological techniques [22]. In this study, we evaluated several risk factors that may be associated with blepharospasm, such as sun exposures, level of urbanization, types of jobs (white-collar or blue-collar workers), sleep disorders, mental disorders (depression, anxiety, and obsessive compulsive disorder), dry eye-related diseases (dry eye and Sjögren’s syndrome), common systemic diseases (hypertension and hyperlipidemia), Parkinson’s disease, and rosacea.

Taiwan could be divided into five regions (Northern, Central, Southern, Eastern, and offshore islands) based on geography, lifestyle, history, and levels of urbanization. Based on different levels of urbanization and types of jobs, we have noticed an interesting distribution of BEB in Taiwan. In our study, the Northern and Central regions reflect a higher urbanization with dominant in white-collar workers and a more stressful lifestyle, a higher incidence of people having blepharospasm was also noted. Southern region and offshore islands are less urbanized, with dominant in blue-collar workers and a relatively non-competitive lifestyle, and a lower incidence of BEB was noted. As now we have learned that some features are known to aggravate blepharospasm such as watching television, reading, and stress [29]. Recently, as computer vision syndrome (CVS) has also become a popular topic, a study by Bali et al. [30] found an increased rate of blepharospasm in those with increased computer use. We postulate that eye muscle straining such as using computer, doing paper work, and watching television which are considered mundane work for white-collar workers, can aggravate blepharospasm. In our study, we hypothesize that the more urbanized regions with more white-collar work, the more likely people will develop blepharospasm.

A systematic review of the relationship between mental health and dystonia found that 12% to 71% of patients with dystonia suffer from depression and anxiety over the course of a lifetime [20]. In our study, which involved a large population of patients with a specific diagnosis of blepharospasm, those with depression, anxiety, and obsessive-compulsive disorder seemed to have an increased risk of developing blepharospasm. Furthermore, among the female population, there appeared to be a stronger association between mental disorders and blepharospasm compared to the male population. Additionally, jobs that are more mentally challenging, such as professional workers, managers, teachers and office workers, were found to be associated with blepharospasm compared to more physical labor-intensive jobs. Although the pathophysiology and neuroanatomy of blepharospasm have not yet been elucidated, there is mounting evidence that blepharospasm may represent a network disorder causing a discoordination of sensory and emotional processing, motor planning, and motor execution, resulting in neuropsychiatric problems [31–34].

A systematic review by Hwang et al. [23] reported that 40 to 70% of patients with focal cranial dystonia (blepharospasm and neck cervical dystonia) have disturbed sleep. Outcomes of our study confirmed this association between sleep disorders and blepharospasm. One hypothesis is that sleep disturbance stems from the same malfunctioning brain regions as those for movement, such as the basal ganglia [23]. Another hypothesis is that psychological distress serves as a mediator for sleep disturbance, leading to an increased risk of developing blepharospasm as described earlier [21]. Our study demonstrated that sleep disorder is highly related to the blepharospasm in both female and male groups, compatible with previously reported literature.

In a study by Conte et al., [35] about 40–60% of patients complain of ophthalmological symptoms (burning, grittiness or dryness) before or at the onset of blepharospasm [35], and a significant association has been reported between blepharospasm and anterior ocular segment diseases such as blepharitis and keratoconjunctivitis [15]. In our study, we found a significant association between dry eye-related diseases (dry eye and Sjögren’s syndrome) and blepharospasm. The association was independent of age, gender, and other risk factors discussed in the study. We specifically excluded patients with anterior ocular segment diseases and ocular surface disorders one month prior to the diagnosis of blepharospasm to avoid confounding effect. In a study by Khodadoust et al.[36], it takes six to seven days for scraped corneal epithelium to regenerate and adhere under fluorescein staining. Furthermore, under examination of histologic sections, it takes up to three weeks for scraped cornea to become indistinguishable from normal cornea. Therefore, we estimated a one-month healing process for corneal injuries and excluded patients with ocular surface disease within one month of the diagnosis of BEB. We hypothesize that, initially, blinking compensates for a dry eye-induced corneal irritation and acts as a protective mechanism and reduces tear film break-up [37]. However, in individuals predisposed to blepharospasm, the nervous system may lose its ability to regulate adjustment in the trigeminal circuits, causing a maladaptation of the “checking system”, creating involuntary spasms of lid closure and trigeminal hyperexcitability [27, 38]. This hypothesis is strengthened by a recent longitudinal study by Conte et al. showing a link between increased blinking and blepharospasm due to an abnormal blink reflex recovery cycle causing the development of ocular orbicularis spasms [39].

Horiuchi et al. [40] compared the risk factor profiles between hemifacial spasm and blepharospasm. Both groups had increased occurrences of hypertension and hyperlipidemia. Hyperlipidemia was found to have a significant association with blepharospasm in our study, more so in the male population than the female population. Although the exact pathophysiology is still unknown for BEB, evidence points to a neural network problem involving certain areas of the brain, causing an involuntary spasm of the facial muscles [31]. Hyperlipidemia is a metabolic disorder underlying atherosclerosis, cerebrovascular disease, and fatty liver disease [41, 42]. Neuroimaging has shown an inverse relationship between triglyceride levels and cerebral blood flow, likely affecting the structural and functional role of the central nervous system [43, 44]. Furthermore, many studies demonstrated that acute stress elicits an increase in the concentration of total cholesterol and low-density lipoprotein (LDL) cholesterol [45–48]. Steptoe et al. also found an association between lipid responses to acute mental stress and fasting serum lipid levels [49]. While further research is needed to clarify the exact mechanism behind the association between blepharospasm and hyperlipidemia, we hypothesize that a stressful lifestyle or job may cause hyperlipidemia, which in turn results in decreased cerebral blood flow impairing areas of the brain that overlap with the pathophysiology of blepharospasm.

Previous reports regarding the link between Parkinsonism and blepharospasm have been controversial–some note blepharospasm as being the most common form of focal dystonia in Parkinsonism [50], while others suggest a lack of relationship between the two. Although our sample size in this area is extremely small, we did find that blepharospasm is more prevalent among patients with Parkinsonism, more so in females. This is interesting as Parkinsonism is of male predominance. This could be due to the nature of BEB with more female in prevalence causing a bias in the result and also, too small of a sample size in general. Again, the pathophysiology of blepharospasm is still unclear, but many lines of research point to the involvement of structural lesions in the basal ganglia, similar to Parkinsonism and other forms of dystonia [50].

A recent report by Khan et al.[9] suggests a causal relationship between BEB and hemifacial spasm with rosacea. In our study, we also found a significant association between BEB and rosacea, especially in the female population. Khan et al. proposed a possible shared immune-inflammatory pathway involved in both facial dystonia and rosacea, but further investigation is needed to elucidate this association [9].

There are several strengths to our study. First, all the data came from the NHIRD, which is a nationwide, population-based, and expanded database including nearly all Taiwanese citizens. This database contains every claims data recorded electronically, minimizing recall bias and ensuring the utmost accurate information. Second, blepharospasm has historically been analyzed as part of focal dystonia due to its scarce occurrence; rarely has the diagnosis examined on its own. In this study, we isolated the diagnosis of blepharospasm in a specific population to determine its associated risk factors. Third, to exclude any secondary cause of blepharospasm and focus only on BEB, we conducted a strict protocol to eliminate any known source of brain injury or lesions. In addition, we excluded patients with ocular surface disease one month prior to being diagnosed with blepharospasm to specifically examine the relationship between dry eye-related diseases and blepharospasm.

There are also some limitations to our study. First, the possibility of a coding error is inherent in any study using claims data. Though diseases, especially the rare ones, are generally diagnosed and coded by specialists and subspecialists, we can only pull diagnostic codes from the database without reviewing patients’ actual medical records or images. Cases may be over- or under-estimated, and the accuracy of diagnoses may vary depending on the visiting physicians. Second, although Blepharospasm is a focal dystonia, it can present as part of a segmental dystonia. However, we did not further distinguish the different subtypes of dystonia in this study as we did not exclude the coding of other types of dystonia at the beginning of our study, which may cause an overestimation of our total cases. Third, we were unable to determine a familial link previously proposed in the literature using this database.

## Conclusion

This study provides a nationwide, population-based data on the incidence of benign essential blepharospasm in Asian adults, which we found to be highly associated with stress-related disease. Higher level of urbanization, white-collar work, sleep disorders, mental health diseases, dry eye-related diseases, Parkinsonism, and rosacea are possible risk factors for BEB.

Future research should focus on elucidating the definitive etiology and pathophysiology of BEB in order to improve the care of blepharospasm through a combination of therapeutic options, lifestyle modification, and preventative measures.
